# Enzymatic Conversion of RBCs by *α*-N-Acetylgalactosaminidase from* Spirosoma linguale*

**DOI:** 10.1155/2019/6972835

**Published:** 2019-05-02

**Authors:** Thomas J. Malinski, Harkewal Singh

**Affiliations:** ^1^Department of Veterinary Pathobiology, University of Missouri, USA; ^2^Department of Chemistry, University of Missouri, USA

## Abstract

*Spirosoma linguale *is a free-living nonpathogenic organism. Like many other bacteria,* S. linguale* produces a cell-associated *α*-N-acetylgalactosaminidase. This work was undertaken to elucidate the nature of this activity. The recombinant enzyme was produced, purified, and examined for biochemical attributes. The purified enzyme was ~50 kDa active as a homodimer in solution. It catalyzed hydrolysis of *α*-N-acetylgalactosamine at pH 7. Calculated K_M_ was 1.1 mM with k_cat_ of 173 s^−1^. The described enzyme belongs to the GH109 family.

## 1. Introduction

Substantial effort has been placed on stabilizing the worldwide blood supply. It is commonplace to see appeals for blood donations because of low levels of supply. Methods to address maintaining a stable blood supply or alternative procedure have been in the research forefront. Enzymatic conversion, perfluorocarbons, hemoglobin-based oxygen carriers, and bonding RBC with polyethylene glycol methodologies have received a great amount of attention. Stem cell research also holds promise; however less work has been pursued in this area due to the political landscape. Each strategy has merit but they are not so without their technical challenges. All of these approaches in concert may eventually play a role in stabilizing the blood supply. The enzymatic conversion of RBC blood type has been a determined pursuit for over three decades. Several research groups have identified a number of enzymes that have the potential to convert RBC blood type. The enzymes targeted to amend the RBC to Type O are *α*-N-acetylgalactosaminidase (A→O) and *α*-galactosidase (B→O). Presently we possess a novel *α*-N-acetylgalactosaminidase from* Spirosoma linguale* that converts Type A RBCs to Type O. The emphasis of this research involves enzymatically converting Type A RBC to O with a recombinant *α*-NAGA originating from* Spirosoma linguale*.* S. linguale* is a common free-living nonpathogenic organism which encodes an *α*-N-acetylgalactosaminidase (*α*-NAGA). *α*-NAGA is an exoglycosidase, within the EC 3.2.1.49 GH109 family classification as described in the carbohydrate-active enzyme database http://www.cazy.org/, which specifically hydrolyzes *α*-N-acetylgalactosamine from the nonreducing terminal sugar of glycoconjugates. The ABO blood group as described by Landsteiner [1] and characterized by Kabat, Watkins, and Morgan [2, 3] is rich in specific glycoconjugates targeted by *α*-NAGA. As indicated the human ABO (types A, B, AB, and O) blood group system, based on the presence or absence of blood group antigens A and B, is mainly differentiated by surface exposed carbohydrate groups designated as ABH. Serologically individuals with type A blood group express Antigen A and antibody to Antigen B. Similarly, individuals with type B blood group express Antigen B and antibody to Antigen A. Blood group AB does not express antibodies; in contrast type O individuals express antibodies to both type A and type B antigens. Therefore, individuals with A antigen produce antibodies to type B blood and vice versa and thus blood transfusions between incompatible blood types leads to immune response and can be fatal.

Biochemically, types A and B red blood cells (RBCs) have different trisaccharide moieties attached to the respective glycoproteins and lipids on their surface. Type A RBCs or type A antigen has GalNAc*α*-1,3-(Fuc*α*-1,2)-Gal*β* trisaccharides that terminate in *α*-1,3-linked N-acetylgalactosamine (GalNAc), whereas the B-antigen consisted of Gal*α*-1,3-(Fuc*α*-1,2)-Gal*β* and terminates in *α*-1,3-linked galactose (Gal) monosaccharide. Type O cells only terminate with Fuc*α*-1,2-Gal*β* disaccharide core, possessing neither GalNAc nor Gal and are nonantigenic. Consequently the major biochemical difference between type A/B and O is the terminal monosaccharide moiety attached to the respective RBCs. Thus, enzymatic removal of terminal GalNAc or Gal represents an attractive strategy to generate universal type O blood.

Goldstein and coworkers used *α* galactosidase from coffee beans to convert type B blood to type O. Unfortunately this enzyme suffered from low turnover rate and was required in copious amounts for effective type O generation.

This in mind *α*-NAGA is also under investigation to enzymatically convert blood group A to O. A number of enzymes have been identified that have attributes to convert RBC blood type [4–16]. Two *α*-NAGAs that demonstrated conversion of Type A_2_ RBCs to blood group O originated from chicken [17] and* C. perfringens* [13]. The enzymatic conversion conditions were not optimal, acidic conditions are required for the chicken enzyme, and the* C. perfringens* enzyme preparation comprised additional glycosidases facilitating the conversion reaction. Both of these enzymes have not been able to demonstrate the enzymatic conversion of Type A_1_ RBCs. To date conversion of Type A_1_ RBCs to O has been the most challenging due to the added complexity of the antigen group [18]. Currently there are relatively few described enzymes identified with the desired characteristics, markedly efficient and active within the blood pH range, and easily generated [19]. Interestingly,* Liu *et al. discovered few efficient enzymes using synthetic substrates that function at neutral pH. Their study showed that nature is perhaps endowed with several naturally occurring enzymes capable of converting type A and type B blood. However, due to complicated biochemistry of type A antigens, up to 60 mg of enzyme was needed for efficient conversion. Keeping this in mind, we initiated our efforts in identifying and exploring the catalytic properties of an *α*-NAGA enzyme to enzymatically convert type A blood to type O blood. In this report, we describe the expression, purification, and characterization of the *α*-NAGA from* S. linguale*. These studies evaluated and characterized this *α*-NAGA to determine the enzyme's optimal conditions (pH, temperature, buffer effects, ionic strength, and specific activity) for cleaving the terminal *α*-N-acetylgalactosamine carbohydrate from type A RBC. This work also estimated an optimal *α*-NAGA concentration per unit of packed RBC, evaluated the feasibility of *α*-NAGA for converting RBC to a universal donor state, and evaluated the effects on the RBC following the *α*-NAGA conversion.

## 2. Materials and Methods

*Spirosoma linguale *#33905 was purchased from the American Type Culture Collection, Rockville, Maryland. Chromatography columns (HisTrap, Q Sepharose, and Zorbax GF-250), SDS-PAGE materials (gels, buffers, and protein markers), QuickChange® Site-Directed Mutagenesis Kit (Stratagene), Western Blot materials (Nitrocellulose Bio Trace™ Pall Life Science, buffers, and developing reagent), BCA and Bradford protein determination solutions, BSA standards, restriction enzymes, the competent cells* E. coli* CD41 (DE3), and TEV protease were obtained from Fisher Scientific, Waltham, Massachusetts. Chromatography and assay reagents and microbiological media were obtained from Fisher Scientific, Waltham, Massachusetts, or VWR Scientific, Radnor, Pennsylvania. Glycosidase conjugates and gel filtration molecular weight markers were obtained from Sigma-Aldrich Company, St. Louis, Missouri. DNA primers were obtained from Integrated DNA Technologies, Coralville, Iowa. The pCR®-Blunt vector and the competent cells* E. coli* DH5*α* and BL21 (DE3) and gel chromatography column were obtained from Invitrogen, Carlsbad, California. The pKA8H vector was obtained from the University of Missouri. Gene amplification was completed using an Eppendorf Mastercycler® pro thermal cycler, Hauppauge, New York. Protein purification was accomplished with an ÄKTAprime™ plus chromatography system from GE Healthcare, Piscataway, New Jersey. Gel chromatography was performed on a Shimadzu LC-10 system, Columbia, Maryland. Protein and enzyme assays were performed on a FLUOstar Omega plate reader from BMG LABTECH, Cary, North Carolina. Fluorescent tagged antibodies were obtained from Santa Cruz Biotechnology, Santa Cruz, California. Flow cell cytometry was performed on an Attune® Acoustic Focusing Cytometer, Carlsbad, California.

### 2.1. Cloning and Mutagenesis

*S. linguale α-NAGA.* The entire coding sequence of locus Slin 6637 was cloned from* S. linguale*. DNA primers were designed to incorporate* Nde*1 and* BamH*1 restriction enzyme sites for amplification of the target gene as described below. 

*S. linguale α-NAGA Primers*

*pKA8H Forward Primer. *CCCGGG**CATATG**CCTTCGCTGTACACGACCGCTCAG

*pKA8H Reverse Primer.* CCCGGG**GGATCC**TCAGTACACGTCGGTAAGGCCAAAGATGGG

The* S. linguale nag* gene was amplified by PCR with the following cycling parameters 94°C for 60 seconds, 52°C for 60 seconds, and 72°C for 60 seconds increasing 10 seconds per cycle for 35 cycles and then maintained at 4°C following completion. The amplified gene products were initially ligated into pCR®-Blunt vector overnight in a water bath incubated at 16°C. The ligation product was transformed into* E. coli* DH5*α* and plated on LB Agar containing kanamycin (40 *μ*g/mL) and then incubated overnight at 37°C. Following incubation, a single colony was picked and grown in LB media supplemented with 40 *μ*g/mL kanamycin. These cultures were incubated at 37°C overnight by constant shaking at 250 rpm in an incubator-shaker for further plasmid preparation. DNA was isolated, double-digested with* Nde*1/*Bam*H1 restriction enzymes, and cloned into pKA8H vector. Briefly, the pKA8H vector codes for an N-terminal 8×His affinity tag and a tobacco etch virus protease site. The cloned genes were confirmed by sequence analysis by the University of Missouri DNA Core Facility.

The* Sl α*-NAGA pKA8H plasmid was transformed into the expression strain* E. coli* BL21(DE3) or CD41(DE3) and plated on LB Agar containing kanamycin (50 *μ*g/mL) and then incubated overnight at 37°C as described. In addition a site-directed mutant of* S. linguale α*-NAGA, in which the purported residue protonates the leaving group in conjunction with NAD^+^, His225, was modified to Alanine (H225A), designed for the purpose of evaluating enzyme activity. 

*S. linguale α-NAGA H225A Mutation Primers*

*Forward Primer*. GATCTCTACCCCACG**GCC**GGTCTGGGGCCGGTG

*Reverse Primer.* CACCGGCCCCAGACC**GGC**CGTGGGGTAGAGATC

The *α*-NAGA mutant was amplified by PCR using the following cycling parameters 94°C for 30 seconds, 55°C for 60 seconds, and 68°C for 7.5 minutes for 20 cycles and then maintained at 4°C following completion. The mutation was introduced into the aforementioned plasmid using the QuickChange® Site-Directed Mutagenesis Kit. The cloned genes were confirmed by sequence analysis by the University of Missouri DNA Core Facility.

### 2.2. Protein Expression and Purification

The* Sl α*-NAGA (cloned into pKA8H vector) plasmid was transformed into the expression strain* E. coli *BL21(DE3) and plated on LB Agar containing kanamycin (40 *μ*g/mL) and then incubated overnight at 37°C. Protein expression was carried out using Studier's autoinduction method [24]. Briefly, single colonies of the transformants were picked to inoculate four 1000 mL cultures made of 1% (w/v) tryptone, 0.5% (w/v) yeast extract, 50 mM phosphate, 50 mM ammonium chloride, 5 mM sulfate, 2 mM magnesium, 1x trace minerals, 0.5% glycerol, 0.05% glucose, 0.2% lactose, and 50 *μ*g/mL ampicillin. The culture was incubated at 37°C with constant shaking at 300 rpm overnight. Cells were harvested approximately 20 hours later by centrifugation at 4°C and 2100 x g and resuspended in 20 mM Tris 150 mM NaCl pH 7.5 or 20 mM sodium phosphate 0.5 M NaCl pH 7.0. The cell pellet was frozen at -20°C for later use. Frozen cells were thawed at 4°C followed by disruption using sonication or French press. Unbroken cells and debris were removed by centrifugation at 4°C for 60 min at 31,000 x g. Additional cellular debris was removed by ultracentrifugation at 4°C for 60 min at 183,960 x g. Supernatant was loaded onto an immobilized metal-ion affinity chromatography column (Ni^2+^-charged HisTRAP, GE Healthcare) using 20 mM Tris 0.15 M NaCl pH 7.5 (buffer A). The protein was eluted using buffer A supplemented with 1 M imidazole. Fractions were pooled and combined with TEV protease (1 mg TEVP for 40 mg of *α*-NAGA) and 1 mM Tris(3-hydroxypropyl)phosphine (THP). Samples were incubated for 8 hours at 20°C and then dialyzed for at least 20 hours in buffer A. Dialyzed protein was loaded onto Ni^++^ immobilized column and tag free enzymes were collected in the flow through. Thus purified SlNAGA was dialyzed into 20 mM Tris pH 7.5 overnight at 4°C. Upon dialysis the protein was aliquoted into thin-walled PCR tubes, quick-frozen in liquid nitrogen, and stored at -70°C. Protein concentration was estimated using bicinchoninic acid (BCA Protein Assay Reagent), Thermo Fisher (Rockford, IL) [25] or the Bradford assay (Bradford Ultra™ Coomassie-based protein quantitation method), and Expedeon (Harston, Cambridgeshire) [26]. Analytical size exclusion chromatography was performed to assess the quality and oligomeric state of the protein.

### 2.3. Enzyme Activity Assay

To assess the enzyme activity and determine the preferred synthetic substrate, a preliminary colorimetric screen was established. Briefly, 80 *μ*L of 20 mM sodium phosphate pH 7.0, 10 *μ*L each of eleven substrates (see below), and 10 mM and 10 *μ*L of *α*-NAGA (Ec and Sl separately) were incubated at 37°C for 30 minutes. At the end of 30 minutes the reaction was quenched using 500 mM glycine pH 10.0 and corresponding samples were measured at 410 nm using a BMG FLUOstar Optima plate reader. Substrates tested included 4-Nitrophenyl conjugates of N-acetyl-*α*-D-galactosaminide, *β*-D-glucopyranoside, *α*-D-galactopyranoside, *α*-L-arabinopyranoside, N-acetyl-*α*-D-glucosamine, N-acetyl-*β*-D-galactosaminide, *α*-D-glucopyranoside, *α*-D-mannopyranoside, *β*-D-galactopyranoside, *α*-L-fucopyranoside, and N-acetyl-*β*-D-glucosamine. OD at 410 nm was used to assess enzyme's relative preference for substrates. Determination of the Michaelis-Menten constants was completed with 4-Nitrophenyl N-acetyl-*α*-D-galactosaminide. The assay utilized 80 *μ*L of 20 mM sodium phosphate pH 7.0 or 7.4, 10 *μ*L of the substrate dilution, and the automated addition of 10 *μ*L of diluted enzyme. Assays to determine the pH optimum of the enzyme for 4-Nitrophenyl N-acetyl-*α*-D-galactosaminide were performed in 20 mM sodium phosphate in the pH range of 6.0-8.0, 50 mM sodium acetate in the pH range of 3.0-6.0, or 100 mM bicine pH 9.0. The assays were performed as previously described using the appropriate buffer solution and substrate and enzyme dilution. Assays to determine temperature response of the enzyme for 4-Nitrophenyl N-acetyl-*α*-D-galactosaminide were performed in 20 mM sodium phosphate pH 7.4 using a temperature range of 20-45°C in increments of 5°C. The assays were performed as previously described using the buffer and substrate solution at the desired temperature with the automated addition of diluted enzyme. Product inhibition assays were performed to determine the effect of N-acetylgalactosamine accumulation. The assays were performed at room temperature (~22°C) in 70 *μ*L of 20 mM sodium phosphate pH 7.0 buffer, 10 *μ*L of the substrate dilution, 10 *μ*L of N-acetylgalactosamine at concentrations of 2.5, 5.0, or 10.0 mM, and the automated addition of 10 *μ*L diluted enzyme.

### 2.4. Chromatography Methods

Sample analysis by gel filtration chromatography was done as follows: approximately 500 *μ*L of purified protein sample was pipetted into a chromatography vial. The chromatography vials were loaded onto a sample tray and placed in the autoinjector. The sequence used for data acquisition included a mixture of five proteins (standard) plus samples from each recombinant protein. The proteins in the standard solution included *β*-amylase, alcohol dehydrogenase, albumin, carbonic anhydrase, and cytochrome C. A Shimadzu LC-10A system was used to do the protein analysis. The system was equipped with a Zorbax GF-250, 4-micron particle size, 4.6 X 250 mm (internal diameter X length) column for assays. The column was maintained at a temperature of 30°C. A TBS (50 mM Tris 150 mM NaCl) pH 7.4, mobile phase flowing at 1.0 mL per minute, was used during the analyses. A UV-Vis detector set at 280 nm wavelength was used to detect the proteins. Chromatographic data were collected with TotalChrom software version 6.2.1.0.104.0104. The molecular mass determination of unknown proteins was made by comparing the ratio of Ve/Vo for the protein in question to the Ve/Vo of protein standards of known molecular mass (Ve: elution volume and Vo: void volume). A calibration curve was generated by plotting the logarithms of the known molecular mass of each protein standard versus their respective Ve/Vo value.

### 2.5. RBC Enzyme Conversion Methods

The initial proof of concept experiment to evaluate enzyme activity* in vivo* was set up as follows: 1 mL of human RBCs in glycine buffer (200 mM pH 6.8) [19] with 100 *μ*g of enzyme was maintained at room temperature (~22°C). These samples were evaluated with standard ABO typing solution. Conversion of RBC followed many of the experiments that were performed during the* in vitro* characterization of the enzyme(s).* In vivo *evaluation of the enzyme(s) included enzyme dose titration, kinetic activity, buffer system, PCV, pH, temperature, product inhibition, and reverse enzyme activity. Blood groups A_1_ and A_2_ RBC were used in the enzyme dose titration and temperature experiments; the remaining experiments were completed with A_2_ RBC. With the exception of the PCV experiment, each test was conducted with 20-30% PCV. All experiments were completed with 0.5 or 1 mL of sample. All samples (except kinetic activity samples) were inverted to mix every 15 minutes to minimize RBC aggregation with 10 *μ*L aliquots removed at 1 and 2 hours and placed in 200 *μ*L of PBS to discontinue enzyme activity. Samples were inverted to mix prior to each aliquot collection for the kinetic activity experiment. Several buffer solutions were evaluated, glycine, alanine, lysine, and PBS. Glycine, alanine, and lysine were tested at 150-300 mM concentrations with 50 mM increments. Phosphate buffer was evaluated at 5 mM; in addition phosphate buffer was evaluated with added NaCl concentrations (ionic strength) ranging from 0 to 20 mM at 5 mM increments. Each assay used 100 *μ*g of enzyme maintained at room temperature. A 250 mM glycine solution was used to evaluate enzyme activity at various pH. The solutions were pH adjusted and ranged from 5.5 to 8.0 at 0.5 pH increments. Each assay used 100 *μ*g of enzyme maintained at room temperature. Samples containing 25, 50, 75, and 100 percent PCV were tested to evaluate the impact of red cell concentration on enzyme activity. Each assay used 100 *μ*g of enzyme maintained at room temperature. An enzyme kinetic experiment composed of samples collected at 0, 1, 5, 10, 15, 30, 45, 60, 75, 90, 105, and 120 minutes after addition of *α*-NAGA was completed with 100 *μ*g of enzyme. Enzyme dose titration experiments were completed with varying *α*-NAGA concentrations at both refrigerated (~ 4°C) and room (~ 22°C) conditions. Enzyme concentrations tested were 0, 1, 5, 10, 20, 25, 30, 40, 50, 60, 70, 75, 80, 90, 100, 250, 300, 350, 400, 450, 500, or 1000 *μ*g. The product, N-acetylgalactosamine, was utilized at concentrations of 10, 50, or 100 mM and was tested with blood group O to evaluate reverse enzyme activity using 250 *μ*g of protein. Inhibition of enzyme activity was evaluated with blood group A_1_ RBC infused with 500 *μ*g *α*-NAGA. The experiment included a sample under the conditions outlined; a sample that at 1 hr was centrifuged and the supernatant was removed and then replaced with fresh buffer, and a sample had 1X additional buffer added after 1 hr. All samples were evaluated by Flow Cytometry.

### 2.6. Flow Cytometry Method

To determine antigenic conversion of RBC each sample was evaluated by Flow Cytometry after enzymatic conversion. Samples were stained with mouse Anti-H FITC, mouse Anti-A, and goat Anti-mouse PE monoclonal antibodies. The initial staining included 1 *μ*L Anti-H FITC and 1 *μ*L Anti-A with 10 *μ*L RBC and 50 *μ*L PBS. The samples were mixed and incubated for 20 minutes in the dark at room temperature. An additional 200 *μ*L PBS was added at the end of the incubation period and centrifuged for 5 minutes at 1500 RPM. The supernatant was removed and the pellet was washed 2 additional times with 200 *μ*L PBS. 1 *μ*L of the PE antibody was added to each sample with 50 *μ*L PBS mixed and incubated in the dark for 20 minutes at room temperature. The samples were washed as described with a final volume of 200 *μ*L PBS added. 150 *μ*L of each sample was diluted into 3 mL of PBS followed by analysis, 10,000 events collected at a flow rate of 100 *μ*L per minute, on the Attune® Acoustic Focusing Cytometer. Unstained FITC and PE stained compensation samples were analyzed prior to RBC analysis.

### 2.7. RBC Immunological Methods

Nine New Zealand White rabbits were challenged with Types A, O, or ECO-A RBC. Laboratory animals were utilized with approval of the Merial Institutional Animal Care and Use Committee, APS 11-99M-09/12. On Days 0 and 21 each rabbit was challenged as indicated in Table 1. Preparation of challenge material was performed, the day of challenge; Day 0 treatments were emulsified in Freund's adjuvant while Day 21 was emulsified in Incomplete Freund's one.

On Day 0, rabbits were bled for baseline antibodies and immunized with the test antigens in CFA. The rabbits were observed for adverse reactions to the challenge. The rabbits were boosted with the test antigens in IFA on Day 21. Blood samples were collected from the ear on Day 28. All rabbits were anesthetized with acepromazine prior to blood collections. Blood samples were collected into a serum tubes, processed into serum, and kept frozen at ≤20°C. Samples were analyzed against blood groups A and O for reactivity to antibodies by Flow Cytometry. Undiluted serum samples were also analyzed for induced antibody response to* S. linguale α*-NAGA by Western Blot.

## 3. Results

### 3.1. Protein Expression, Purification, and Characterization

*α*-NAGA overexpressions were achieved using the methods by Studier [24] as described. Cells were harvested and the overexpressed enzyme was released by rupturing. Cell debris was removed using two-step centrifugation and resulting supernatant was used for further purification. Both enzymes were purified using two-step purification schemes, that is, Ni^++^-IMAC affinity chromatography followed by ion exchange chromatography. Enzyme activity was measured using the end point assay described above. His tag thus purified enzyme was cleaved using TEVP and subsequent affinity step resulted in high amounts of tag free protein (data not shown). Tag free *α*-NAGA was further purified using ion exchange chromatography. Purified fraction of Tag free *α*-NAGA was injected onto size exclusion chromatography for analysis. *α*-NAGA eluted at column volume corresponding to a molecular mass of 96430 Da which suggested that the enzyme may exist in solution as dimer.

**Figure 1:**
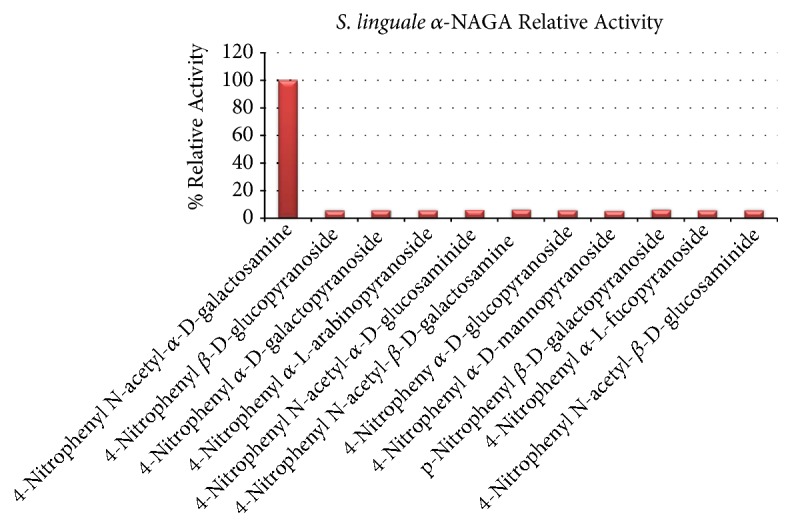
Relative* S. linguale α*-NAGA activity to carbohydrate substrates.

To assess the relative activity of SlNAGA, a semiquantitative enzyme activity assay was performed against several substrates. Specifically, 4-Nitrophenyl N-acetyl-*α*-D-galactosaminide,4-Nitrophenyl *β*-D-glucopyranoside, 4-Nitrophenyl *α*-D-galactopyranoside, 4-Nitrophenyl *α*-L-arabinopyranoside, 4-Nitrophenyl N-acetyl-*α*-D-glucosamine, 4-Nitrophenyl N-acetyl-*β*-D-galactosaminide, 4-Nitrophenyl *α*-D-glucopyranoside, 4-Nitrophenyl *α*-D-mannopyranoside, p-Nitrophenyl *β*-D-galactopyranoside, 4-Nitrophenyl *α*-L-fucopyranoside, and 4-Nitrophenyl N-acetyl-*β*-D-glucosamine were tested using the method described above. SlNAGA exhibited the highest activity against 4-Nitrophenyl N-acetyl-*α*-D-galactosaminide (Figure 1). Furthermore, steady state kinetic parameters of SlNAGA were estimated using 4-Nitrophenyl N-acetyl-*α*-D-galactosaminide substrate. At 20°C, K_m_ is estimated to be 0.63 mM while k_cat_ is 128 /s and catalytic efficiency is 203 (s^−1^mM^−1^). Similar specific activity was demonstrated by* E. meningoseptica* [28, 29] and* C. perfringens* [30]  *α*-NAGA to *α*-N-acetylgalactosamine substrate.

The enzyme assays characterizing *α*-NAGA proteins from* S. linguale* included steady state kinetics with variable substrate(s), pH, temperatures, and buffers. The assays were performed with 80 *μ*L buffer, 10 *μ*L conjugate substrate, and 10 *μ*L enzyme in 96-well plates consisting of 100 *μ*L total volume. A BMG FLUOstar Optima plate reader with a 410 nm reference filter was used to collect data. VisualEnzymics 2010 and/or Origin Pro v8 software was used to calculate the Michaelis-Menten factors.

**Figure 2:**
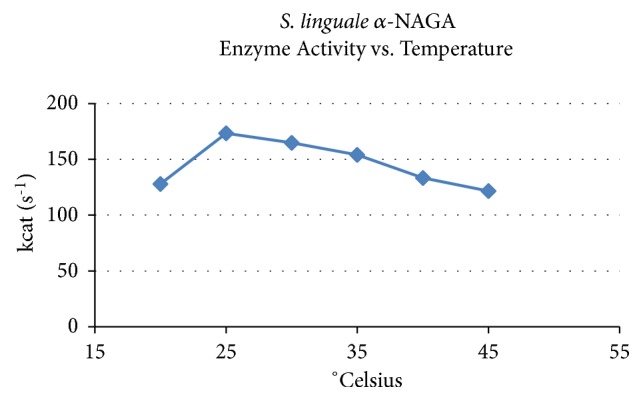
*S. linguale α*-NAGA activity versus temperature.

**Figure 3:**
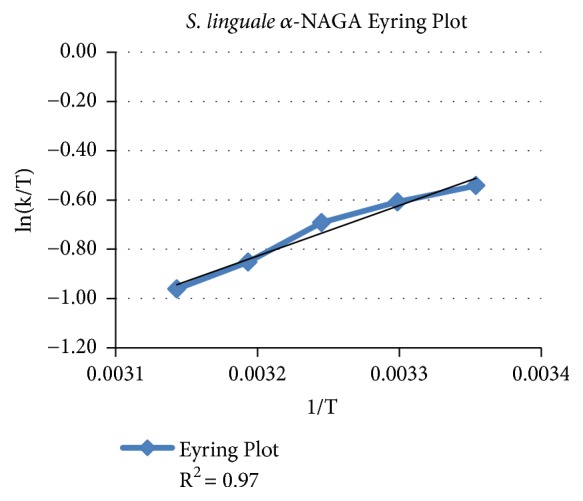
Eyring plot* S. linguale α*-NAGA activity versus temperature.

**Figure 4:**
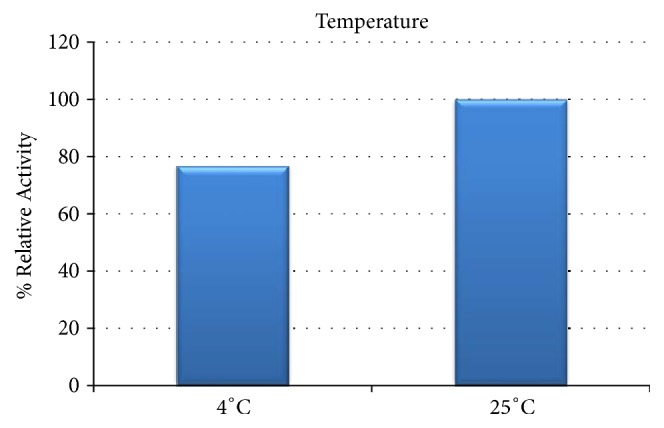
*S. linguale α*-NAGA activity 4°C versus 25°C.

For the purified recombinant protein enzyme activity response to temperature variances is shown in Figure 2. The* S. linguale* enzyme activity was levelled and decreased with elevated temperature. Eyring plot shows that the enzyme has a linear response to temperature change, as shown in Figure 3. The Eyring equation is a theoretical model based on the transition state that describes the temperature dependence of the reaction rate [25].* S. linguale* enzyme activity has a linear response in the direction of lower temperature. To evaluate this response the enzyme activity was compared at 4°C and 25°C. Enzyme activity was approximately 25% lower at 4°C, as shown in Figure 4.

Michaelis-Menten steady state parameters were calculated to evaluate temperature enzyme response characteristics, as shown in Table 2. Assays to determine temperature response of the enzyme for 4-Nitrophenyl N-acetyl-*α*-D-galactosaminide were performed in 20 mM sodium phosphate pH 7.4 using a temperature range of 20-45°C in increments of 5°C. The turnover rate, k_cat_, of the* S. linguale *enzyme, was relatively flat over the temperature range tested. At the same time substrate affinity, K_M_, increased and then declined over the temperature range while the catalytic efficiency, k_cat_/K_M_, remained somewhat constant.

**Figure 5:**
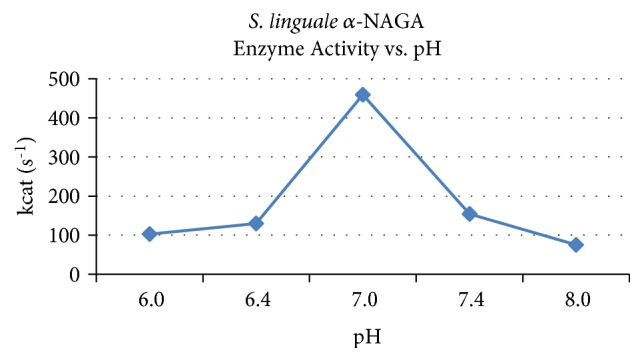
*S. linguale α*-NAGA activity versus pH.

Assays to determine the pH optimum of the enzyme for 4-Nitrophenyl N-acetyl-*α*-D-galactosaminide were performed in 20 mM sodium phosphate in the pH range of 6.0-8.0, 50 mM sodium acetate in the pH range of 3.0-6.0, or 100 mM bicine pH 9.0. Enzyme activity decreased at acidic pH and then peaked to be close to neutral pH and again decreased as pH became alkaline. Enzyme activity of* S. linguale* was the highest approximately pH 7.0, as shown in Figure 5.

Michaelis-Menten steady state parameters were calculated to evaluate pH enzyme response characteristics, as shown in Table 3.* S. linguale* enzyme exhibited increased turnover rate as the optimal pH was achieved with declining rate in the acidic or alkaline range.* S. linguale α*-NAGA substrate affinity, turnover rate, and catalytic efficiency were at the highest levels close to neutral pH. Van Etten has expressed concerns of evaluating pH optimum with conjugated substrates due to effects of the pKa of the leaving group [26]. The pKa of the leaving group of 4-Nitrophenyl N-acetyl-*α*-D-galactosaminide is 7.6. With the leaving group pKa within the pH range of the optimum of the enzyme the value of the conjugate data is uncertain.

**Figure 6:**
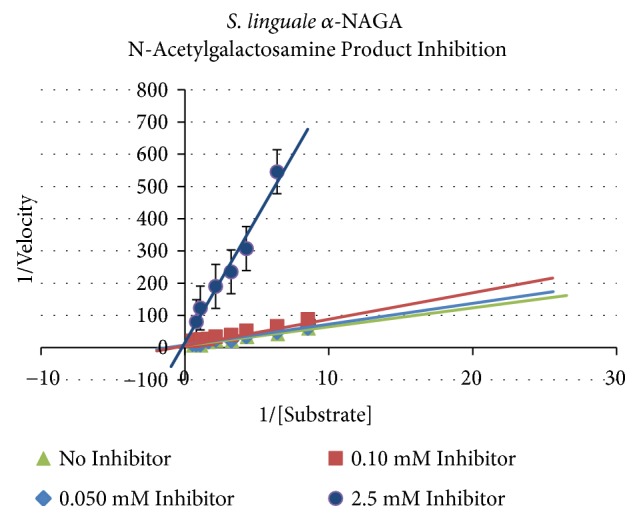
Product inhibition* S. linguale α*-NAGA activity.

Inhibition analyses have been performed on similar *α*-NAGA proteins. Lui et al. showed inhibition of* E. meningoseptica* enzyme activity with several divalent metals, Cu^2+^, Ni^2+^, and Zn^2+^, at concentrations of 1 or 10 mM but EDTA did not impact enzyme activity at these concentrations [19]. Undiluted Adsol solution tested against the* C. perfringens* enzyme was the only solution to lower activity as demonstrated by Hsieh and Smith [9]. Product inhibition does not appear to be evaluated. Because the goal is to use these enzymes to convert RBCs enzyme activity brings about accumulation of product which may impact complete conversion of RBCs. Approximately 0.4 *μ*M/L of N-acetylgalactosamine is present on RBCs. The product, N-acetylgalactosamine, was used to test if increased product generation would inhibit enzyme activity. The inhibition assays were performed at room temperature (~22°C) in 70 *μ*L of 20 mM sodium phosphate pH 7.0 buffer, 10 *μ*L of the substrate dilution, 10 *μ*L of N-acetylgalactosamine at concentrations of 0.05, 0.1, 2.5, 5.0, or 10.0 mM, and the automated addition of 10 *μ*L diluted enzyme.* S. linguale α*-NAGA was affected by product generation. The Lineweaver-Burk plots, in Figure 6, provide an overview of product inhibition. The* S. linguale* enzyme appears to be sensitive to N-acetylgalactosamine concentrations as low as 50 *μ*M. Three inhibition kinetic models, competitive, noncompetitive, and uncompetitive, were evaluated with the kinetic data using VisualEnzymics 2010 to determine the appropriate model to calculate the K_i_. VisualEnzymics allows the user to perform an ANOVA comparing the modeled data to select the correct model procedure. A competitive model was used to calculate the Michaelis-Menten inhibition parameters of* S. linguale α*-NAGA. The calculated parameters, k_cat_ and k_cat_/K_M_, indicate a marked difference compared to pH enzyme activity but to a lesser degree of the temperature data, as shown in Table 4.

**Figure 7:**
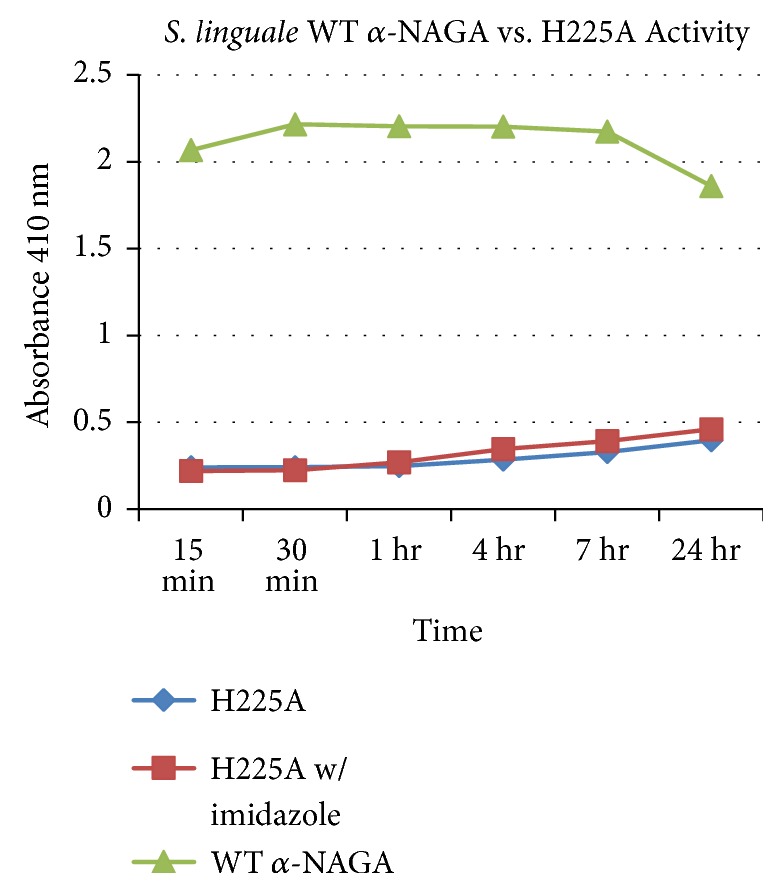
*S. linguale* wild type versus H225A *α*-NAGA activity.

A genetic mutation of the purported histidine cofactor, amino acid 225, to alanine, was introduced to evaluate* S. linguale α*-NAGA activity. Histidine 225 acts in concert with a tightly integrated NAD^+^ cofactor during the hydrolysis reaction of the N-acetylgalactosamine molecule. The mutation H225A eliminated enzymatic activity disrupting the ability of the enzyme to catalyze hydrolysis of N-acetylgalactosamine. Enzymatic activity was monitored at selected intervals comparing the wild type enzyme to H225A with or without supplemental imidazole, as shown in Figure 7. Imidazole supplemented with wild type enzyme was not evaluated because during purification steps it was visually noted that enzyme activity was impeded.

**Figure 8:**
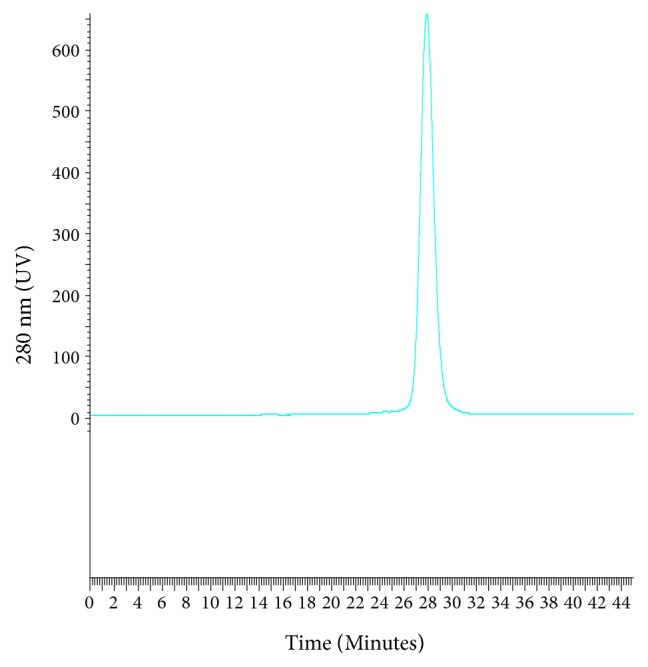
*S. linguale α*-NAGA purity by gel filtration chromatography.

**Figure 9:**
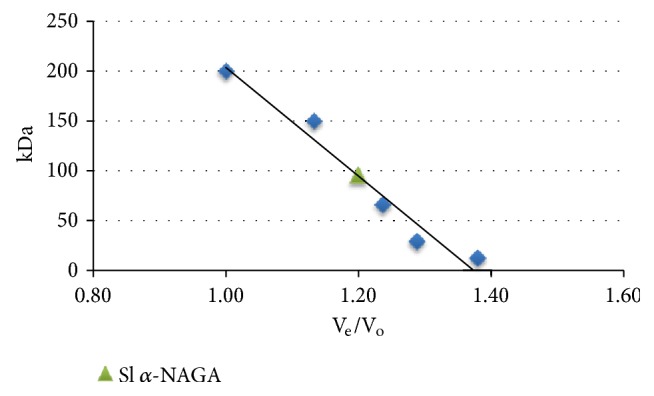
Gel filtration chromatography molecular mass estimation.

Purity of the* S. linguale* enzyme by gel filtration chromatography is shown in Figure 8. The estimated molecular weight of* S. linguale* as determined by gel filtration chromatography, 96,000 Da, is shown in Table 5 and Figure 9.* S. linguale* protein appears to act as dimer under the conditions tested.

### 3.2. RBC Enzyme Conversion Results

**Figure 10:**
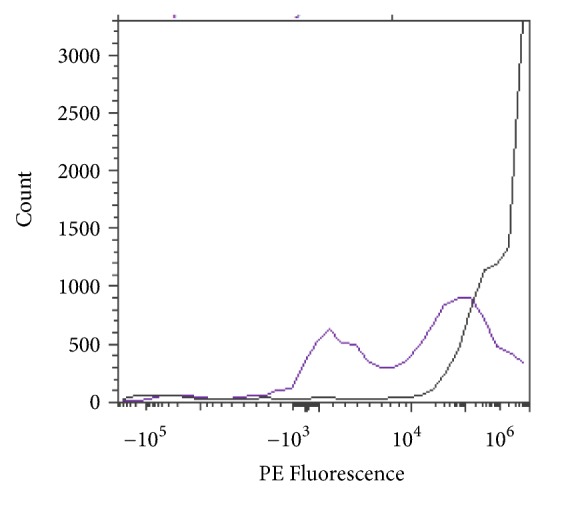
Flow cytometry profile of PE stained blood subgroups A_1_ and A_2_.

RBCs were stained with a fluorescent fluorophore, phycoerythrin (PE), and fluorescein isothiocyanate (FITC), to determine blood group status before and/or after enzymatic conversion. Blood group A is composed of the subgroups A_1_ and A_2_, each of which generates a distinct PE flow cytometric histogram as shown in Figure 10.

**Figure 11:**
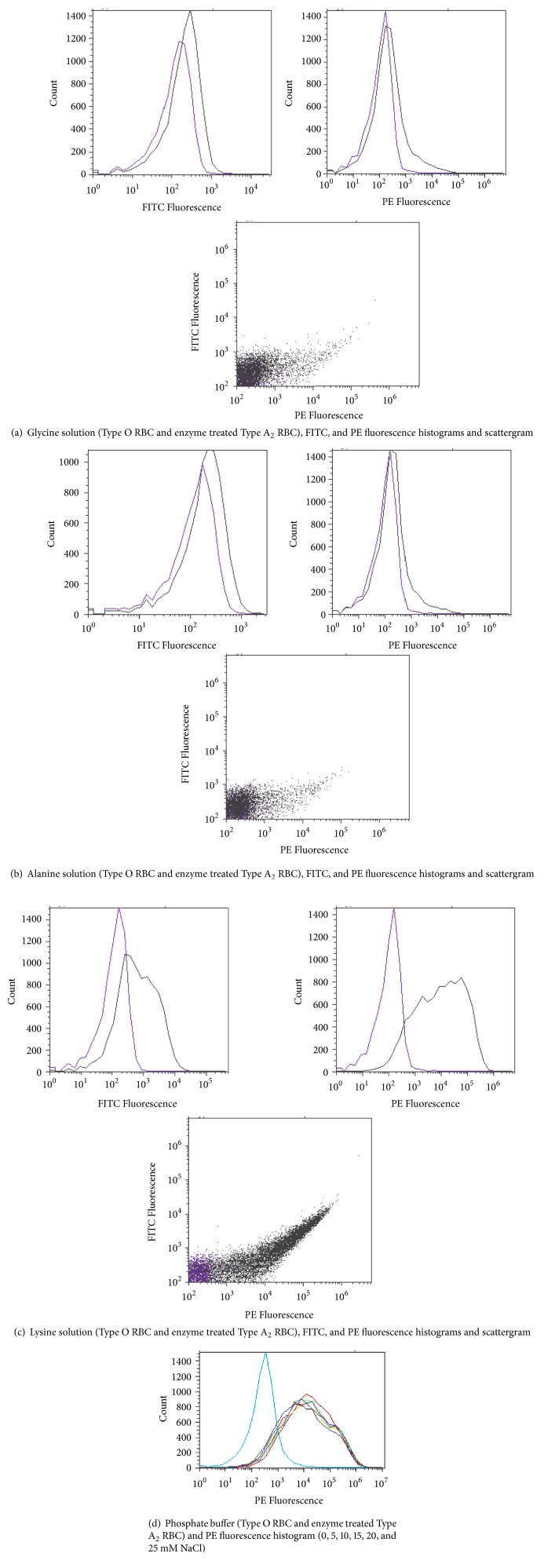
*S. linguale α*-NAGA blood group conversion by solution.

RBCs were subjected to* S. linguale α*-NAGA to determine the conversion of blood group A to O under several conditions, for example, pH, PCV, temperature, and enzyme amount (*μ*g/mL). Four solutions were tested, glycine, alanine, lysine, and phosphate, with varying amounts of NaCl, to determine an optimal conversion solution. Glycine, alanine, and lysine solutions were tested at 150, 200, 250, 300, and 350 mM pH 7.0. The phosphate buffer solution was tested at 5 mM phosphate pH 7.0, with 0, 5, 10, 15, 20, or 25 mM NaCl. The RBC conversion solutions glycine, alanine, and lysine figures are in 250 mM solutions.* S. linguale α*-NAGA was evaluated under these conditions, as shown in Figures 11(a)–11(d). It appeared that conversion of blood group A to O occurred in the glycine and alanine solutions but did not occur in the lysine or phosphate buffer solutions following 1-hour incubation at room temperature.

**Figure 12:**
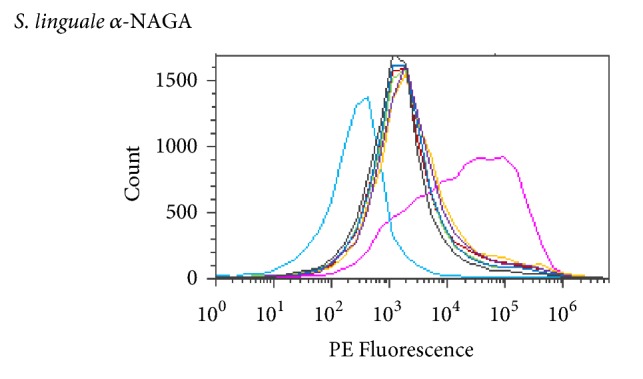
250 mM glycine solution (Type O RBC, Type A_2_ RBC, and enzyme treated Type A_2_ RBC) and PE fluorescence histogram (5.5, 6.0, 6.5, 7.0, 7.5, and 8.0 pH).

Enzymatic activity was determined in glycine and alanine solutions. Optimal pH enzyme activity was tested in 250 mM glycine solution pH 5.5, 6.0, 6.5, 7.5, or 8.0, as shown in Figure 12. RBCs were incubated for 1 hour with* S. linguale α*-NAGA at room temperature.

**Figure 13:**
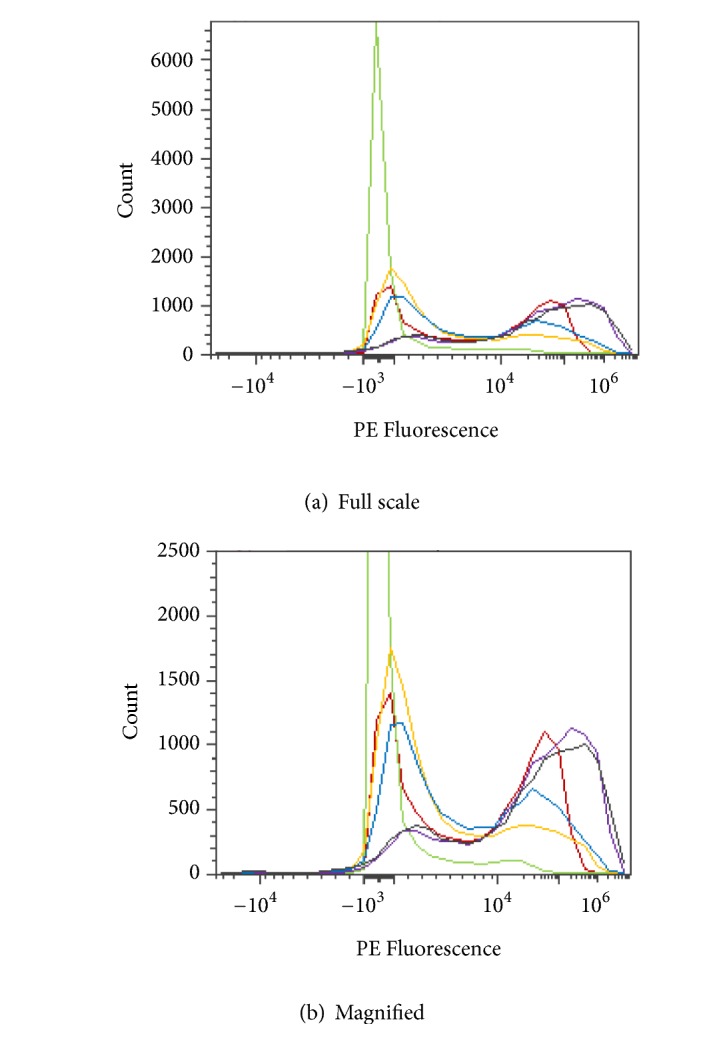
250 mM glycine buffer (Type O RBC, Type A_2_ RBC, and enzyme treated Type A_2_ RBC) and PE fluorescence histogram (100, 75, 50, and 25 PCV).

The effects of packed cell volume, PCV, related to enzyme activity, were tested with* S. linguale α*-NAGA in 250 mM glycine pH 7.0, at room temperature with 25, 50, or 75 percent PCV, as shown in Figures 13(a) and 13(b). No additional solution was added to the 100% PCV sample.

**Figure 14:**
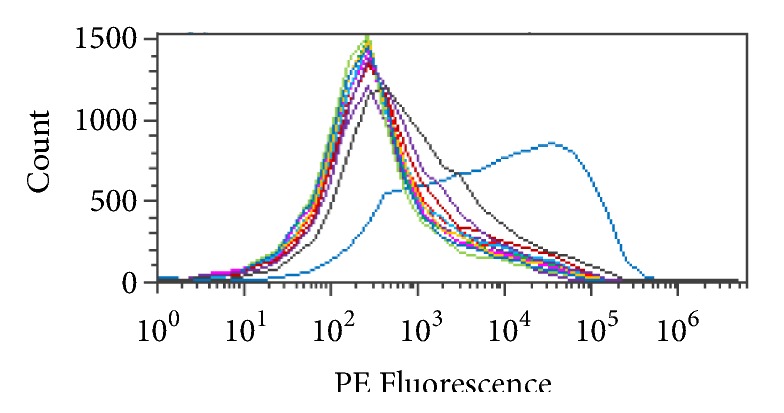
*S. linguale α*-NAGA at room temperature (Type O RBC, Type A_2_ RBC, and enzyme treated Type A_2_ RBC) and PE fluorescence histogram (10, 20, 30, 40, 50, 60, 70, 80, 90, and 100 *μ*g/mL enzyme).

**Figure 15:**
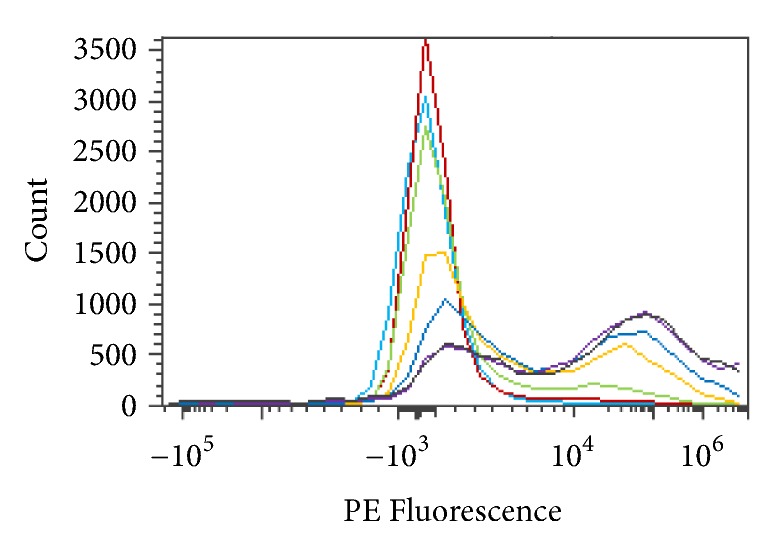
*S. linguale α*-NAGA at room temperature for 1-hour incubation (Type O RBC and enzyme treated Type A_2_ RBC) and PE fluorescence histogram (0, 1, 10, 25, 50, and 100 *μ*g/mL enzyme).

**Figure 16:**
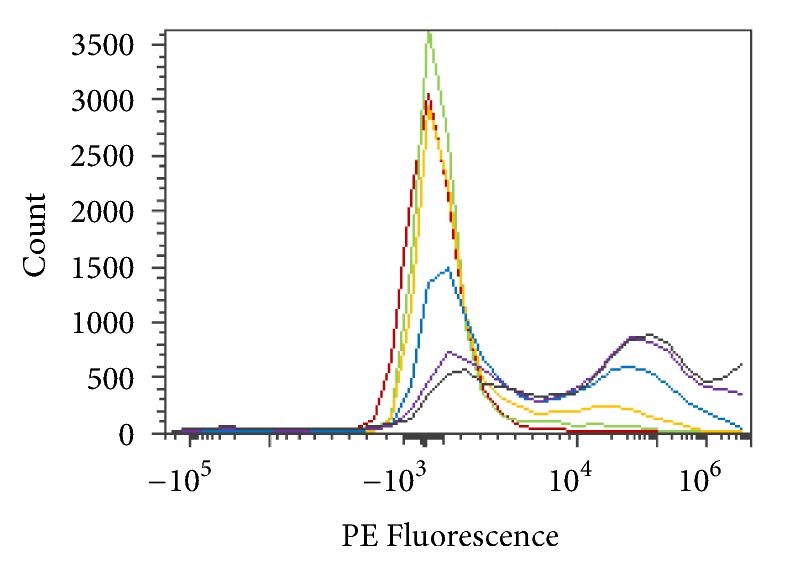
*S. linguale α*-NAGA at room temperature for 2-hour incubation (Type O RBC and enzyme treated Type A_2_ RBC) and PE fluorescence histogram (0, 1, 10, 25, 50, and 100 *μ*g/mL enzyme).

**Figure 17:**
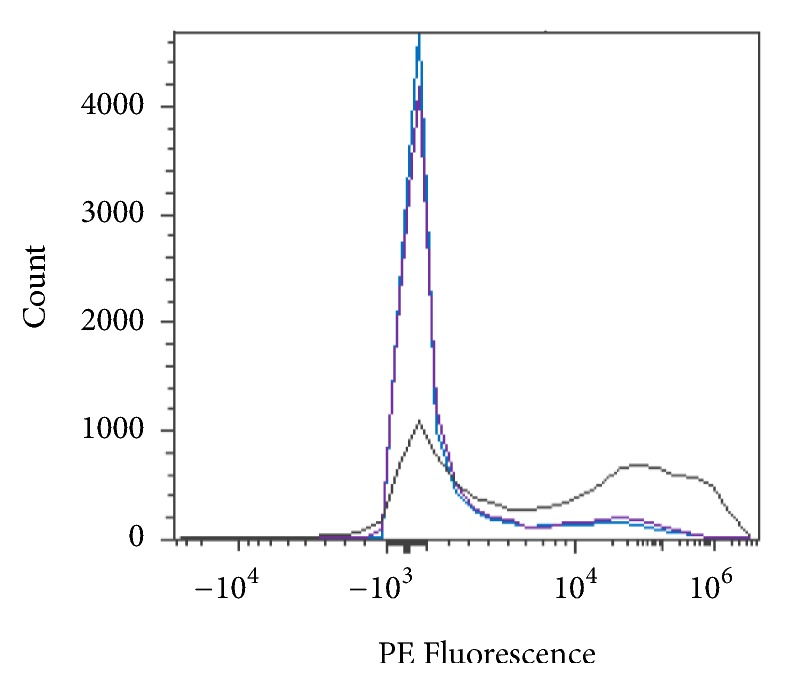
*S. linguale α*-NAGA at refrigerated temperature 2-hour incubation (Type O RBC and enzyme treated Type A_2_ RBC) and PE fluorescence histogram (350 and 400 *μ*g/mL enzyme).

**Figure 18:**
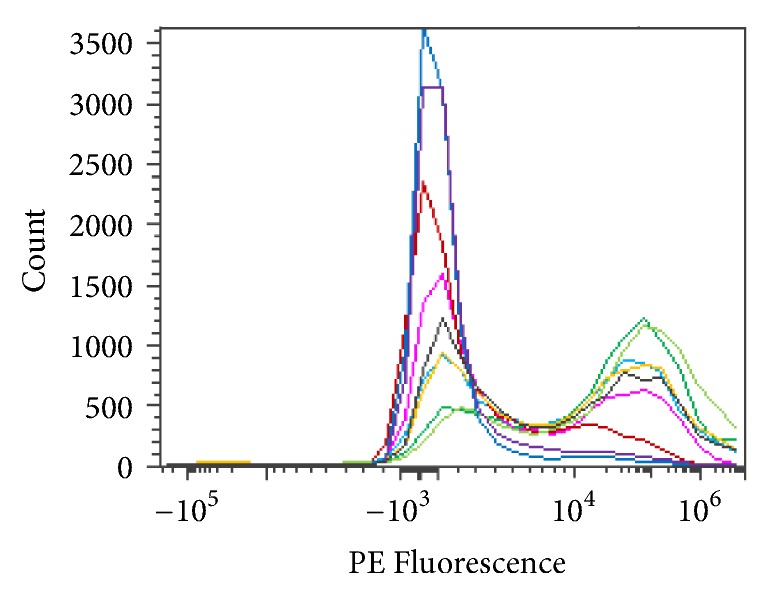
*S. linguale α*-NAGA at room temperature for 2-hour incubation (enzyme treated Type A_1_ RBC) and PE fluorescence histogram (500 *μ*g/mL enzyme).

**Figure 19:**
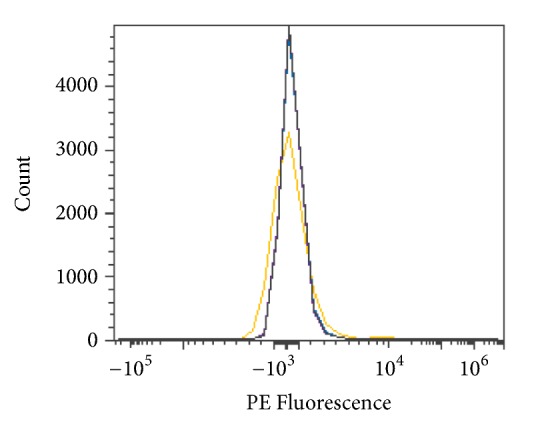
*S. linguale α*-NAGA at room temperature for 2-hour incubation (enzyme treated Type A_1_ RBC) and PE fluorescence histogram (500 *μ*g/mL enzyme). Enzyme conversion without additional steps, enzyme conversion with solution replacement, enzyme conversion with additional solution added, and Type O RBC.

**Figure 20:**
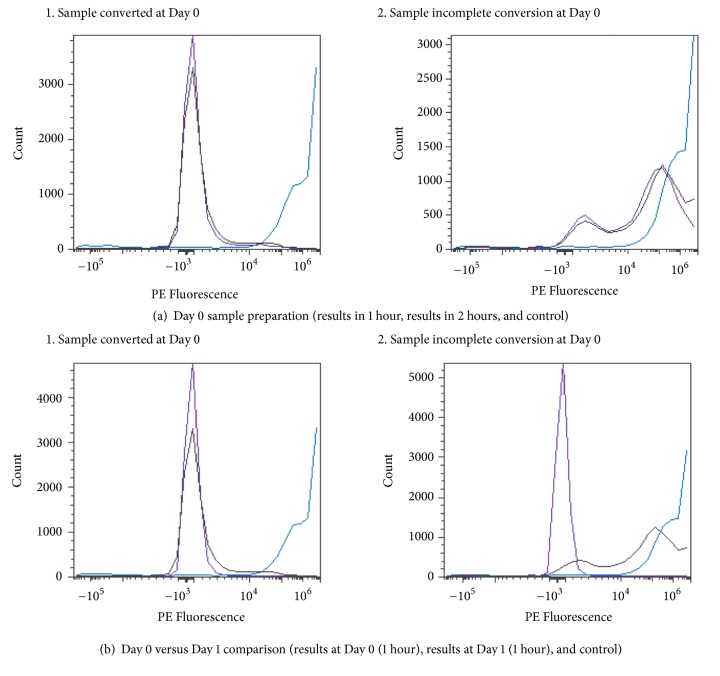
*S. linguale α*-NAGA at room temperature (enzyme treated Type A_1_ RBC) and PE fluorescence histogram (500 *μ*g/mL enzyme).

**Figure 21:**
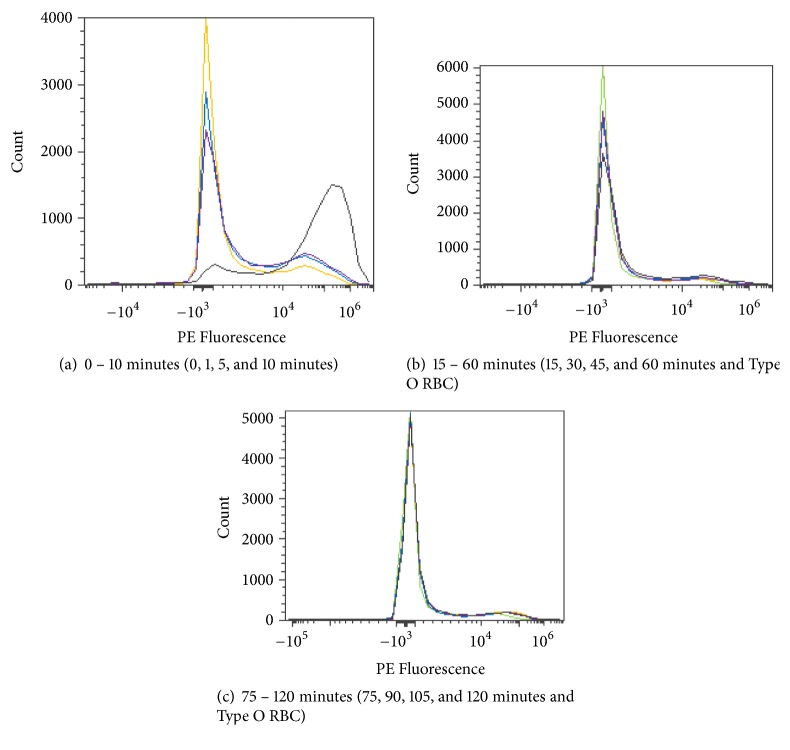
*S. linguale α*-NAGA at room temperature (enzyme treated Type A_2_ RBC) and PE fluorescence histogram (100 *μ*g/mL enzyme).

Various amounts of enzyme were used to determine the optimal level to convert RBCs in solution. The amount of enzyme ranged from 1 to 1,000 *μ*g/mL. A 250 mM glycine pH 7.2 solution at room or refrigerated temperature was used to evaluate enzyme activity. All Figures 14–21 will indicate the enzyme amounts and the testing conditions.* S. linguale α*-NAGA was tested at several enzyme concentrations ranging from 1 to 100 *μ*g/mL of enzyme at room temperature and 100 – 500 *μ*g/mL of enzyme at refrigerated temperature of treated Type A_2_ RBCs. The initial test performed at room temperature with 10 – 100 *μ*g/mL of enzyme indicated conversion of RBCs without any apparent difference in enzyme concentration required, as shown in Figure 14. Additional testing with an enzyme concentration range of 1 – 100 *μ*g/mL of enzyme provided definitive results. At 1 hour of incubation apparent conversion of RBCs appeared between 50 and 100 *μ*g/mL of enzyme, as shown in Figure 15. Allowing 2 hours of incubation resulted in conversion of RBCs with between 25 and 50 *μ*g/mL of enzyme, as shown in Figure 16. At refrigerated temperatures approximately 8 times additional enzyme, 400 *μ*g/mL, is required to achieve conversion of RBCs, as shown in Figure 17. Type A_1_ RBCs have approximately 6 times more antigen sites than A_2_ RBCs, 1.5 versus 0.25 million, respectively. Initial titration of Type A_1_ RBCs with 100 – 500 *μ*g/mL of enzyme at refrigerated temperature did not convert the RBCs to Type O. At room temperature this enzyme titration range yielded mixed results at the 500 *μ*g/mL enzyme concentration, as shown in Figure 18. Several of the samples have the Type A_2_ flow cytometric signature indicating that enzymatic conversion is occurring but is still incomplete. Based on the* in vitro* data* S. linguale α*-NAGA was shown to be sensitive to product accumulation. An experiment to evaluate this potential event compared 3 samples set up as described; with 3 samples after 1-hour incubation the samples were centrifuged with the subsequent supernatant removed and replaced with fresh solution, and with 3 samples after 1-hour incubation additional solution was added effectively diluting the sample by two, as shown in Figure 19. No differences were noted from these two additional treatments. While evaluating the data an unexpected result occurred, on the day glycine buffer was added to the Type A_1_ RBCs excess material was prepared, saved, and used on two consecutive days. This involves the results noted in Figures 18 and 19. These data were compared with the original data collected on Day 0, as shown in Figure 20(a), and the data collected on Day 1 overlaid with Day 0 data, as shown in Figure 20(b). This data suggests that pretreatment with the conversion solution approximately 24 hours prior to blood conversion enhances enzymatic activity.

*In vivo* kinetics of the* S. linguale α*-NAGA was evaluated with Type A_2_ RBCs. The experiment used 250 mM glycine solution pH 7.0, with RBCs at approximately 30% PCV and 100 *μ*g/mL enzyme. Samples were collected at preenzyme treatment (0 min) and 1, 5, 10, 15, 30, 45, 60, 75, 90, 105, and 120 minutes after treatment. Conversion of the Type A_2_ RBCs appears to occur at approximately 15 minutes after enzyme addition, as shown in Figures 21(a)–21(c).

**Figure 22:**
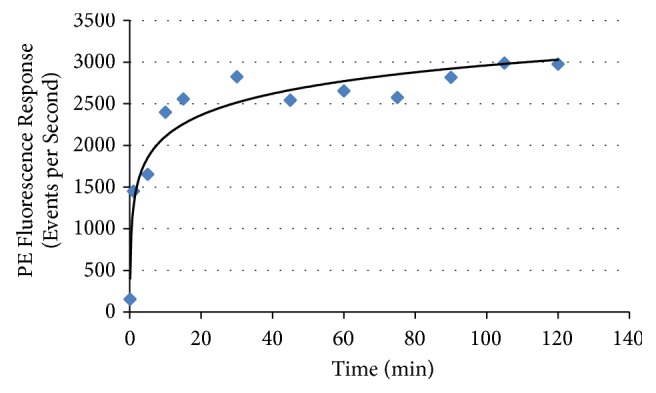
*S. linguale α*-NAGA enzyme conversion of treated Type A_2_ RBCs over time.

Plotting out the fluorescence response (events per second) over time appears to indicate saturation of the enzyme activity during liberation of the *α*-N-acetylgalactosamine sugar from the A antigen, as shown in Figure 22.

**Figure 23:**
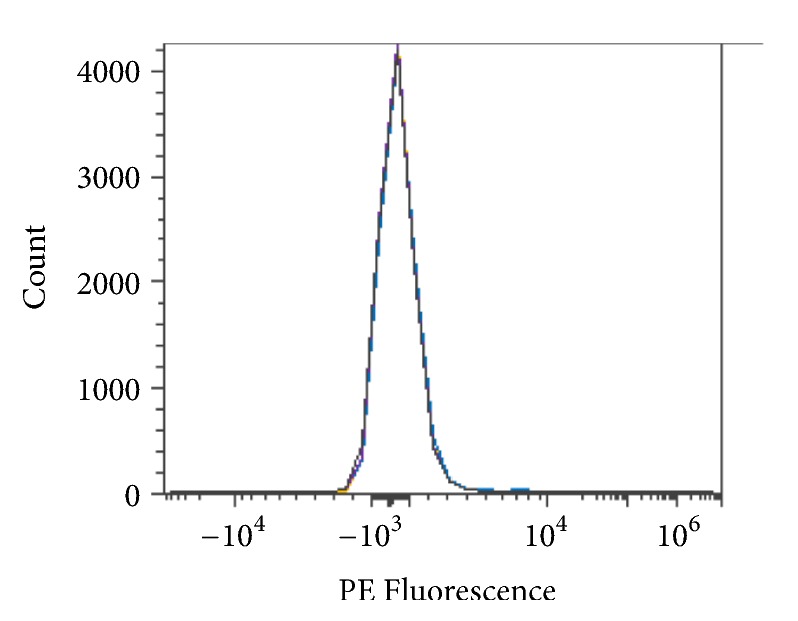
Reverse enzyme activity* S. linguale α*-NAGA at room temperature (enzyme treated Type O RBC), PE fluorescence histogram (250 *μ*g/mL enzyme), 2-hour incubation, and control 0 mM substrate, 10 mM substrate, 50 mM substrate, and 100 mM substrate.

Reverse enzyme activity was tested to determine any residual sugar addition to the antigen complex. Type O RBCs were combined with enzyme, 250 mM glycine solution pH 7.2, and 0, 10, 50, or 100 mM N-acetylgalactosamine and then incubated for 2 hours at room temperature. Conversion to Type A RBCs does not appear to occur in the presence of N-acetylgalactosamine, as shown in Figure 23.

**Figure 24:**
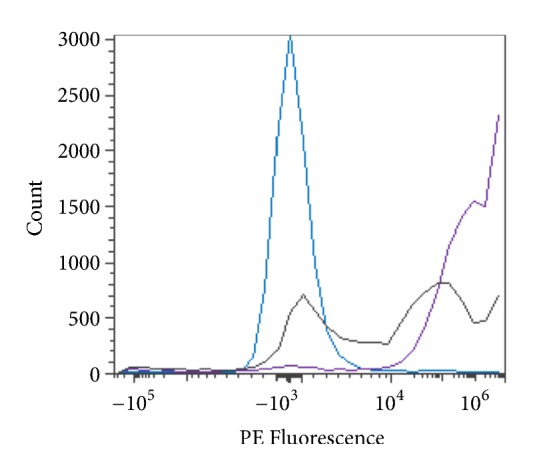
*S. linguale *H225A *α*-NAGA at room temperature (enzyme treated Type A_1_ and A_2_ RBC), PE fluorescence histogram (250 *μ*g/mL enzyme), 2-hour incubation, Type A_2_ RBC, Type A_1_ RBC, and Type O RBC.

*In vitro S. linguale *H225A mutant did not exhibit enzyme activity. The H225A mutant was also tested* in vivo* with Type A_1_ and A_2_ RBCs for the presence of enzyme activity. Type A_1_ and A_2_ RBCs were combined with H225A enzyme and 250 mM glycine solution pH 7.2 and then incubated for 2 hours at room temperature. The H225A mutant does not appear to convert Type A_1_ or A_2_ RBCs to the Type O blood group, as shown in Figure 24.

### 3.3. RBC Immunological Results

Nine New Zealand White rabbits were challenged with Types A, O, or ECO-A RBCs. Laboratory animals were utilized with approval of the Merial Institutional Animal Care and Use Committee, APS 11-99M-09/12. On Days 0 and 21 each rabbit was challenged as indicated in Table 1, in Section 2. Preparation of challenge material was performed, the day of challenge, and Day 0 treatments were emulsified in complete Freund's adjuvant while Day 21 was emulsified in incomplete Freund's.

**Figure 25:**
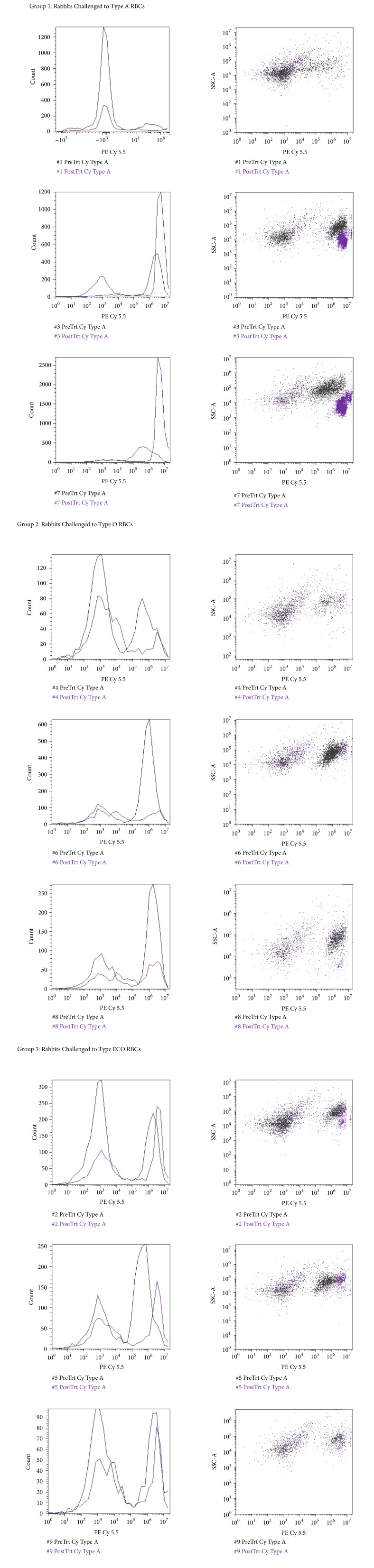
Rabbit antibody response to Type A tetrasaccharide.

**Figure 26:**
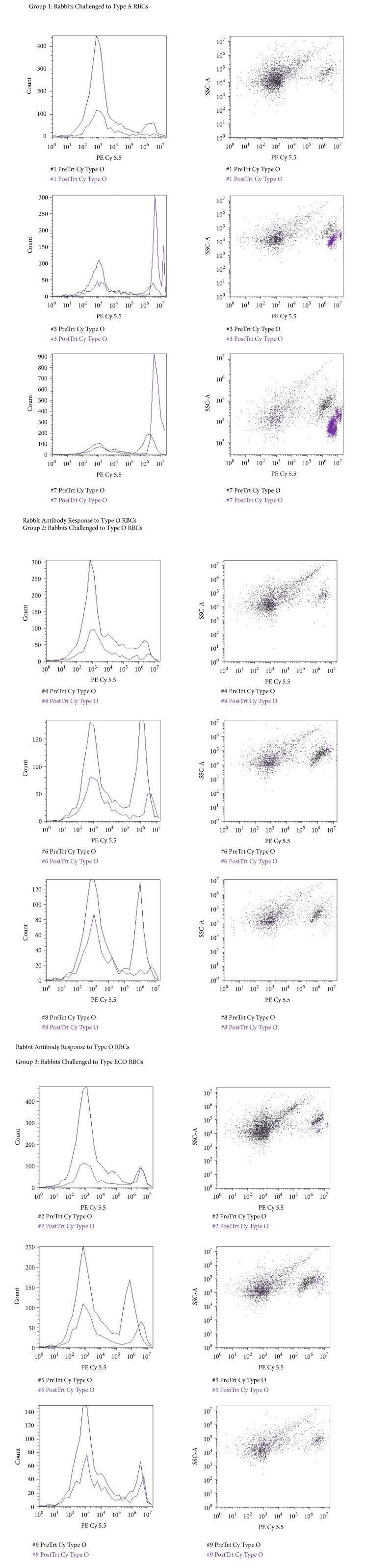
Rabbit antibody response to Type H antigen trisaccharide.

**Figure 27:**
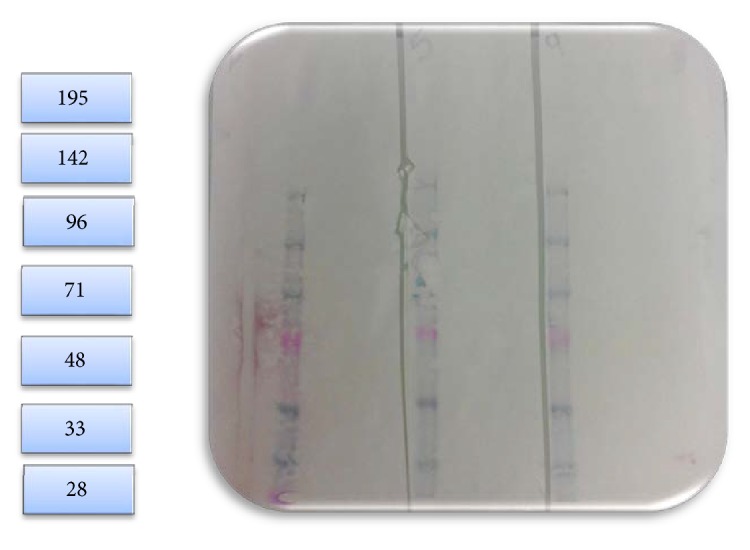
*Western Blot of the S. linguale α-NAGA ECO-A challenged rabbits*. Lanes 1, 3, and 5: protein standard markers (kDa), lane 2: rabbit #2, lane 4: rabbit #5, and lane 6: rabbit #9.

On Day 0, rabbits were bled for baseline antibodies and immunized with the test antigens in CFA. The rabbits were observed for adverse reactions to the challenge. The rabbits were boosted with the test antigens in IFA on Day 21. Blood samples were collected from the ear on Day 28. All rabbits were anesthetized prior to blood collections. Blood samples were collected into serum tubes, processed into serum, and kept frozen at ≤20°C. Samples were analyzed against blood group A tetrasaccharide and H trisaccharide substrates for reactivity to antibodies by Flow Cytometry, as shown in Figures 25 and 26. Two of three rabbits challenged with Type A RBCs not only responded strongly to the A antigen tetrasaccharide but also responded moderately to the H antigen trisaccharide. The rabbits challenged with Type O RBCs did not appear to have an induced response to either antigen. Two of the ECO-A rabbits had a weak-to-low antibody response to the A antigen substrate and all three rabbits responded similarly to the H antigen as the rabbits challenged with Type O RBCs. Serum samples from the ECO-A challenged rabbits were also analyzed for induced antibody response to* S. linguale  α*-NAGA by Western Blot, as shown in Figure 27. There was no apparent induced antibody response to* S. linguale α*-NAGA by the ECO-A challenged rabbits.

## 4. Discussion

**Figure 28:**
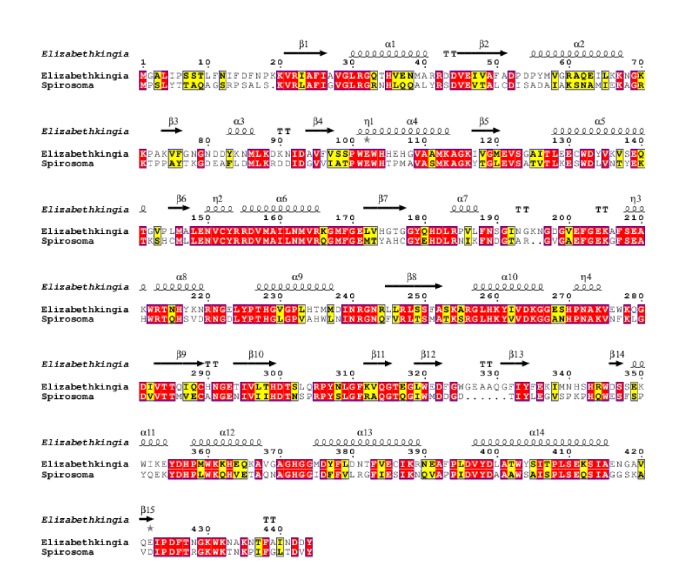
*α*-NAGA sequence alignment* S. linguale *and* E. meningoseptica* [20, 21].

Enzymatic conversion of one blood group to another has been sought for over three decades. A number of enzymes have been identified that have desirable attributes and convert RBC blood types A, B, and AB to O [4–15, 31]. Two *α*-NAGAs that demonstrated conversion of Type A_2_ RBCs to blood group O originated from chicken [17] and* C. perfringens* [13]. The enzymatic conversion conditions were not optimal, acidic conditions are required for the chicken enzyme, and the* C. perfringens* enzyme preparation included additional glycosidases. Both of these enzymes have not been able to demonstrate the enzymatic conversion of Type A_1_ RBCs. To date conversion of Type A_1_ RBCs to O has been the most challenging due to the added complexity of the antigen group [18]. Currently there are relatively few described enzymes identified with the desired characteristics of highly efficient and specific, active within the blood pH range, and being easily generated [19]. The results from this research project are consistent with the identification of *α*-NAGA from* S. linguale* that has excellent properties to convert blood Type A to O. Our data suggest that the* S. linguale α*-NAGA enzyme has potential use as a tool to convert type specific RBCs to universal RBCs. This has been demonstrated by the specificity of the enzyme for the target substrate, high enzyme activity using various buffers, temperatures, and optimal activity at a neutral pH. Sequence alignment shows that* S. linguale α*-NAGA has 52% identity to the* E. meningoseptica α*-NAGA, as shown in Figure 28.

As determined by the relative identity and protein sequence of the investigated *α*-NAGA,* S. linguale* enzyme falls within the GH109 family classification. The GH109 family involves a NAD^+^ dependent hydrolysis mechanism of *α*-N-acetylgalactosamine as described by Koshland [32]. The glycoside hydrolase family 109 uses a mechanism that requires an NAD^+^ cofactor, which remains tightly bound throughout catalysis. The reaction begins with an anionic transition state with elimination and redox steps. There is an initial oxidation of the hydroxyl group attached to carbon 3 by the enzyme-bound NAD^+^ cofactor. This causes the acidity of carbon 2 to increase whereby the enzymatic base, for example, histidine 225, removes a proton. An *α*,*β*-unsaturated intermediate is formed followed by the addition of water at the anomeric carbon. The ketone at C3 is reduced generating the hydrolyzed sugar resetting the enzyme. This reaction sequence was determined by NMR studies, kinetic isotope properties, equilibrium studies, X-ray crystallography, and UV/Vis spectrophotometry [33, 34]. The* S. linguale α*-NAGA purported hydrolytic reaction involves histidine 225 in conjunction of the NAD^+^ cofactor as the H225A mutation abrogates enzymatic activity* in vitro*, conjugated substrate, and* in vivo* RBCs.

Initial physicochemical substrate specificity of the enzyme with the reagent *α*-N-acetylgalactosamine was highly selective compared to eleven additional substrates. Similar specific activity was demonstrated by* E. meningoseptica* [19, 27] and* C. perfringens* [35]  *α*-NAGA to *α*-N-acetylgalactosamine substrate. Optimal enzyme activity was demonstrated by* S. linguale* in the pH range of 6.0 - 8.0; well within the range RBCs are stored. Comparison of Michaelis-Menten kinetic parameters in Table 6 illustrates the diverse activity and substrate affinity of this enzyme class. The catalytic efficiency of the* S. linguale α*-NAGA indicates approximately a 25% greater efficiency to the* E. meningoseptica α*-NAGA.

*S. linguale* exhibited a linear response to temperature. The enzyme originates from a mesophilic organism where the response of* S. linguale α*-NAGA activity moderately decreased with increasing temperature.* S. linguale α*-NAGA demonstrated optimal enzyme activity at 25°C. When assessed at 4°C the* S. linguale *enzyme activity decreased approximately to 25% with Type A_2_ RBCs.

Using a phosphate buffer pH 7.0, as a base to relate the effect on enzyme activity, solutions composed of 50 mM acetate, 10 mM citrate, 10 mM tris, and 500 mM EDTA appeared to reduce relative enzyme activity. A glycine solution, used in studies by Liu et al. [19] and Yu et al. [27], appeared to reduce enzyme activity* in vitro* compared to the phosphate solution. Liu et al. showed inhibition of* E. meningosetica* enzyme activity with several divalent metals, Cu^2+^, Ni^2+^, and Zn^2+^, at concentrations of 1 or 10 mM but EDTA did not impact enzyme activity at these concentrations [19]. Undiluted Adsol solution tested against the* C. perfringens* enzyme was the only solution to lower activity as demonstrated by Hsieh and Smith [9]. Product accumulation was tested for enzyme inhibition during this research project. The* S. linguale α*-NAGA indicated a concerning level of competitive inhibition during product accumulation. The reaction mechanism proposed by Liu et al. [19] outlined that H228 was involved with the NAD^+^ cofactor during the enzyme hydrolysis reaction. The H228, as aligned with the* S. linguale* sequence, was compared to H225. Histidine 225 was mutated to an alanine to assess enzyme activity. The H225A mutation resulted in the production of an inactive enzyme when compared to the wild type recombinant *α*-NAGA.

Conversion of Type A_1_ RBCs to blood group O has proved to be difficult. The ability to convert Type A_2_ and B RBCs has been known for over three decades. The difficulty with Type A_1_ RBCs stands out because of the number of antigen sites, the linkage type of the carbohydrate chains, and the number of carbohydrate repeats of the antigen of this blood group compared to Type A_2_ or B RBCs.  Table 7 provides a picture of the difference between Types A_1_ and A_2_. The difference illustrated is only of the approximate antigen number of the two Type A subgroups. Using the* in vitro* turnover rate, k_cat_ (s^−1^), of the two enzymes of this research and two closely related enzymes, the significant difference to convert Type A RBCs to blood group O is astounding.

The *α*-NAGAs from* S. linguale* and* E. meningoseptica* have both demonstrated the capacity to convert Type A RBC to blood group O in this research and by two groups Liu et al. [19] and Yu et al. [27], respectively. The* E. meningoseptica* enzymes used [19, 27] had very similar steady state Michaelis-Menten kinetic parameters. An alignment of the protein sequences is identical with the exception of four amino acids at positions 160G>V, 174H>R, 426F>S, and 434V>A. The major variance between works reported by Liu et al. and Yu et al. is the enzyme amount required to convert RBCs. For instance, Liu et al. show that to convert 200 mL of packed RBCs enzyme required for Type A_1_ 60 mg is needed and for A_2_ 15 mg on the other hand Yu et al. reported the enzyme requirements as 3.0 mg for A_1_ and 0.8 mg for A_2_, a 95% difference. The estimated concentration of* S. linguale α*-NAGA required to convert 200 mL of packed Type A_1_ RBCs is 80 mg and for A_2_ 20 mg. There may be an explanation for the difference but it is not evident.

Evaluation of four solutions demonstrated that glycine or alanine could equally serve as buffer of choice to convert RBCs. Phosphate buffer with varied ionic strength was a unsuitable buffer selection to convert RBCs for either enzyme. Smith and Walker [36] showed that a glycine solution was a superior selection to enzymatically convert RBCs. Glycine and alanine are known to disrupt the negative charged environment around the red cell membrane [37].

**Figure 29:**
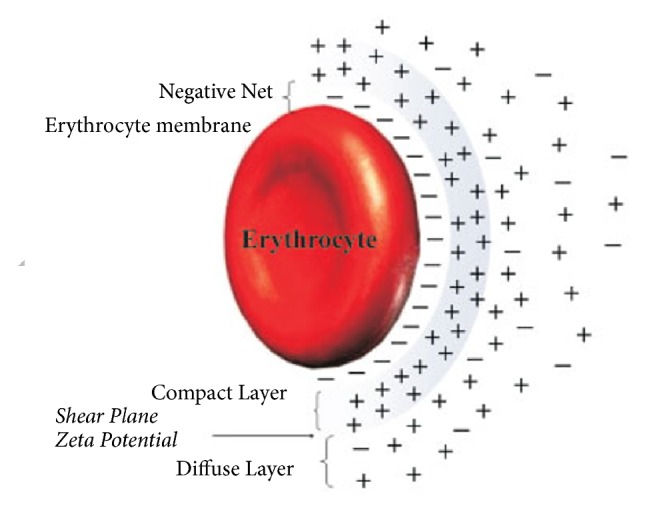
Illustration of the zeta potential of RBCs [22].

Erythrocytes (negative charges) in suspension causing a rearrangement of charges through the formation of two ionic layers that generate an electrical potential difference between them are called the zeta potential, as shown in Figure 29.

**Figure 30:**
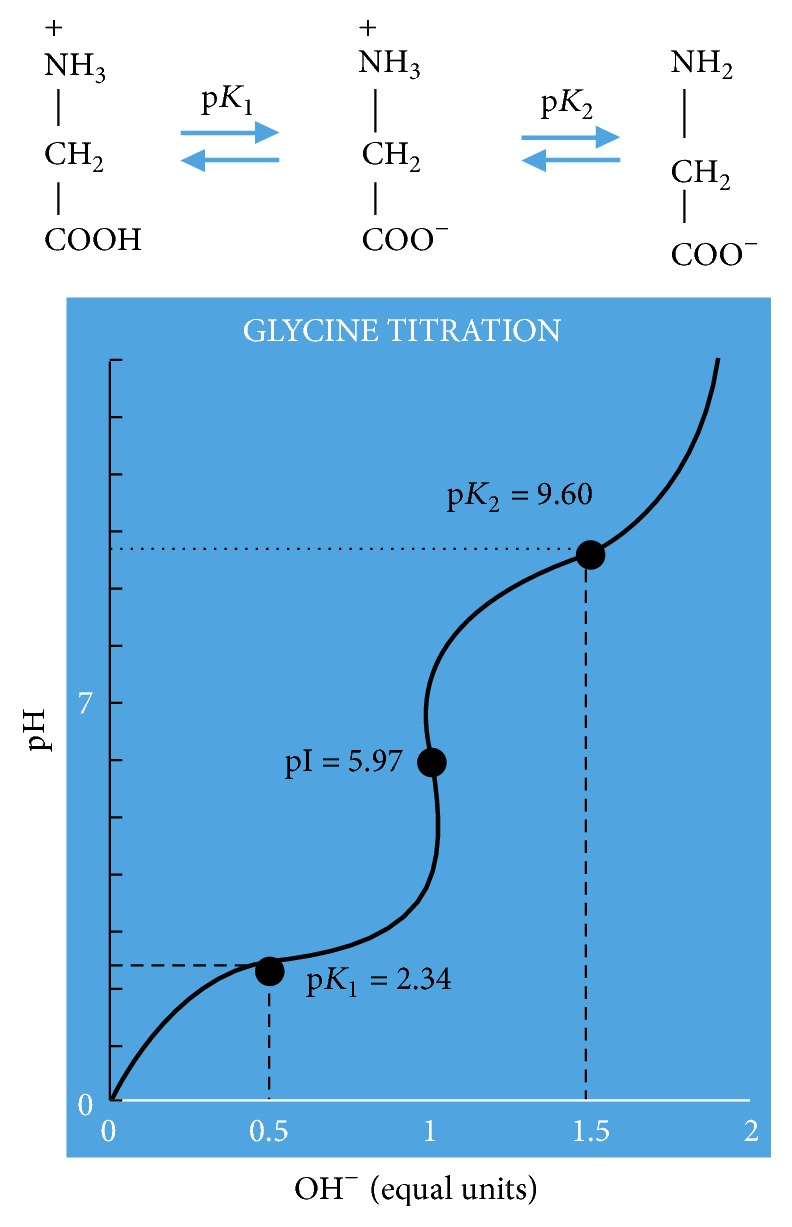
Illustration of the titration of glycine [23].

Glycine and alanine both serve as potentiators reducing the zeta potential around the RBC enhancing enzyme activity. The titration of glycine provides us with an understanding of why it serves as a potentiator, as shown in Figure 30. Because glycine and alanine are zwitterions within the pH range of 4-8, they have a net zero charge. This characteristic equally allows both amino acids to neutralize both positive and negative charges. For this same reason lysine and phosphate solutions, having either a positive or a negative overall charge, do not create an environment for the enzyme to interact with RBCs because of the inability of reducing the zeta potential.

This modified environment appears to improve the *α*-NAGA RBC interface leading to the hydrolysis of *α*-N-acetylgalactosamine. An observation made during the conversion of RBCs involved pretreating cells in glycine buffer the day prior to adding enzyme. This led to additional enzyme efficiency specifically for Type A_1_ RBC conversion. Due to the reduced zeta potential by glycine additional care of the blood sample is required when using these potentiators because agglutination of RBCs is augmented. Jan and Chien showed the effect of altering the charged environment around the RBC impacted aggregation [38]. Due to the effects of glycine* S. linguale α*-NAGA exhibited significant enzyme activity* in vivo* across a broad pH range, 5.5-8.0. The enzyme demonstrated efficient activity at both room and refrigerated temperatures but requires approximately 10X more enzymes when used at 4°C. Packed cell volume approaching 50% has a negative impact on enzyme activity. A packed cell volume range used in this research was 20-35%.* In vitro* testing suggested that product accumulation would be competitive during enzyme activity. Competitive inhibition due to product accrual was not noted during any experiment. Experimental data suggested the potential for reverse enzyme activity when evaluating Flow Cytometry results collected at 1 and 2 hours. Testing with Type O RBCs and *α*-N-acetylgalactosamine indicated that conversion to Type A RBC did not occur.* In vivo* testing with the mutant H225A did not account for any conversion of Type A RBCs.

Stability of the RBCs after enzymatic conversion is paramount if it is to advance beyond the research laboratory environment. On the surface the conversion process of RBCs seems to be minimal cleaving the terminal *α*-N-acetylgalactosamine. Nevertheless this is a multiple step procedure that may place stress on the RBC. This research project inspected the stability of enzyme converted RBCs. RBC count, hemoglobin concentration, and % hematocrit showed statistical significance between Type A RBCs and enzyme converted cells. Visual hemolysis was observed in the enzyme converted RBC samples. The primary cause of hemolysis appears to be linked to the centrifugation steps in which the RBCs were subjected. The Type A unconverted cells did not undergo centrifugation when prepared. Graphically the data from the three groups, Type A and cells converted at room or refrigerated temperature, appear similar. Clinically the RBC samples may behave not differently. Goldstein showed that enzyme converted Type B cells behaved and survived similarly to unconverted RBCs [12]. Vosnidou et al. incurred similar results as this research when comparing converted and nonconverted RBCs [14]. Kruskall et al. noted that hemoglobin concentration decreased from approximately 7.4 g/dL after enzyme conversion which was thought to be related to the washing steps following conversion of RBC [39]. Within the same study RBC survival was found to be similar comparing converted and nonconverted blood. Additional studies should be conducted to fully evaluate the presence or absence of cellular stress to converted RBCs.

Typically foreign host proteins induce an immune response when injected into the system. The* S. linguale α*-NAGA did not appear to induce an immunological reaction in the rabbit model when challenged with enzyme converted RBCs. A follow-up blood transfusion study will need to be accomplished to provide a definitive indication whether* S. linguale α*-NAGA has immunological properties. Two studies using *α*–galactosidase from coffee beans triggered an immune response after transfusion of enzyme converted blood in Rhesus monkeys and humans [15, 39]. Each of the studies did not observe any adverse events relating to this antibody response. The two studies involved single transfused units of blood. Transfusion of multiple units of blood over time has not been investigated in subjects to evaluate for a potential increased immune response and/or reaction.

Infusing larger amounts of the enzymes to evaluate immunological/toxicological concerns is currently sparse in the literature. A 150 *μ*g/kg level of *α*–galactosidase from coffee beans has been demonstrated to be safe in Rhesus monkeys [15]. Zhang determined that this concentration represented approximately 2500X by body weight (*μ*g/kg) safe concentrations of enzyme. The Zhang study indicated that approximately 10 ng/mL of enzyme remained after conversion and washing of the RBCs. An approximate 1X dose of enzyme based on the Zhang study would equate to 60 ng/kg of *α*-NAGA. Notwithstanding the immune response to residual enzyme following conversion of the RBCs the response of blood transfusion reactions begets a higher level of concern to the recipient.

Nine New Zealand White rabbits were challenged with Types A, O, or ECO-A RBCs. Each rabbit was challenged on Days 0 and 21 according to group assignment. Baseline and postchallenge blood samples were collected to determine induced antibody response. The samples were analyzed against blood group A tetrasaccharide and H trisaccharide substrates for reactivity to ABO blood antibodies by Flow Cytometry. Two of the rabbits challenged with Type A RBCs not only responded strongly to the A antigen tetrasaccharide but also responded moderately to the H antigen trisaccharide. The rabbits challenged with Type O RBCs did not appear to have an induced response to either antigen. Two of the ECO-A rabbits had a weak-to-low induced antibody response to the A antigen substrate and all three rabbits responded similarly to the H antigen as the rabbits challenged with Type O RBCs. This initial experiment provided valuable mixed data but additional studies to evaluate transfused enzyme converted blood to test subjects need to be completed to determine the extent of an antibody response. There has been limited experimental work to determine the effects of enzyme converted transfused blood.

In 1991, Lenny et al. successfully transfused a small volume of B blood converted to O using a *α*-galactosidase from green coffee beans [40]. In 1994 and 1995, Lenny et al. successfully transfused full unit(s) of converted B to O blood into volunteers [41, 42]. No clinical reactions were observed in the volunteers, nor were there abnormal laboratory test results from blood samples taken during the study. In 2000, Kruskall et al. successfully transfused converted B to O blood into 21 patients [39]. No adverse reactions were noted in any of the patients. Red blood cell survival was comparable between patients transfused with converted versus nonconverted RBCs in all but two patients; one patient had gastrointestinal bleeding at the time of the transfusion and another patient's serum was incompatible with the transfused red blood cells. Posttransfusion monitoring of the patients noted that 5 of 19 developed an increased anti-B titer. These achievements not only have provided a step forward but also have raised additional questions. Better understanding of the anti-B immune response and the cross-matching compatibility issue with serum or plasma are pivotal to this aim. As stated these studies used *α*-galactosidase from green coffee beans. Currently additional immunological data that may exist is not available regarding enzymes under investigation.

## 5. Summary

This research program characterized the physicochemical attributes of* S. linguale α*-NAGA. The initial process to convert RBCs with the* S. linguale α*-NAGA was established. The experimental testing provided confirmation to the conversion of RBCs. Quality and storage stability of the converted RBCs was exhibited. Initial data was produced to evaluate the immunological properties of* S. linguale α*-NAGA. The concept and ground work has been established to convert RBCs with* S. linguale α*-NAGA.

## Figures and Tables

**Table 1:** Rabbit challenge treatment schedule.

Group	Vaccine	Dose volume	Route of administration	Frequency of administration	No. of animals
1	Type A blood group	500,000 RBC/~1 mL	Subcutaneous	2 doses, Days 0 and 21	3
2	Type O blood group	500,000 RBC/~1 mL	Subcutaneous	2 doses, Days 0 and 21	3
3	Type A-ECO blood group	500,000 RBC/~1 mL	Subcutaneous	2 doses, Days 0 and 21	3

**Table 2:** Temperature Michaelis-Menten steady state kinetics.

Enzyme	Temperature °C	*μ*mole N-Acetylgalactosamine/s	K_M_ (mM)	k_cat_s^−1^	k_cat_/K_M_ (s^−1^mM^−1^)
*S. linguale* *α*-NAGA	20	0.14 ± 0.005	0.63 ± 0.05	128	203
	25	0.19 ± 0.007	1.07 ± 0.07	173	163
	30	0.18 ± 0.012	1.09 ± 0.12	165	152
	35	0.17 ± 0.014	0.94 ± 0.15	154	163
	40	0.14 ± 0.012	0.67 ± 0.12	133	198
	45	0.13 ± 0.006	0.79 ± 0.08	122	154

**Table 3:** pH Michaelis-Menten steady state kinetics.

Enzyme	pH	*µ*mole N-Acetylgalactosamine/s	K_M_ (mM)	k_cat_s^−1^	k_cat_/K_M_ (s^−1^mM^−1^)
*S. linguale* *α*-NAGA	6.0	0.11 ± 0.01	0.50 ± 0.08	103	206
	6.4	0.14 ± 0.02	0.89 ± 0.18	130	145
	7.0	0.50 ± 0.05	0.67 ± 0.12	459	686
	7.4	0.17 ± 0.01	0.20 ± 0.04	154	765
	8.0	0.08 ± 0.01	0.13 ± 0.03	75	579

**Table 4:** Michaelis-Menten single substrate inhibition kinetics.

Enzyme	*µ*mole N-Acetyl-galactosamine/s	K_M_ (*µ*M)	K_i_ (mM)	k_cat_s^−1^	k_cat_/K_M_ (s^−1^mM^−1^)
*S. linguale* *α*-NAGA	0.15 ± 0.006	0.80 ± 0.069	0.18 ± 0.024	138	173

**Table 5:** Gel filtration chromatography molecular mass estimation.

*S. linguale α*-NAGA						
	(Da)	V_e_	V_e_/V_o_			
*β*-Amylase	200000	24.51	1.00			
Alcohol dehydrogenase	150000	27.76	1.13			
Albumin	66000	30.30	1.24			
Carbonic anhydrase	29000	31.57	1.29			
Cytochrome C	12400	33.81	1.38		Monomer	Dimer
				Est (Da)	Calculated (Da)	Calculated (Da)
*S. linguale α*-NAGA		29.39	1.20	96000	48215	96430

**Table 6:** *α*-NAGA Michaelis-Menten kinetic factors.

*α*-NAGA source	K_M_ (mM)	k_cat_ (s^−1^)	k_cat_/K_M_ (s^−1^mM^−1^)	% Difference k_cat_/K_M_*∗*
*Elizabethkingia meningoseptica *[19]	0.077	9.8	128	78
*Elizabethkingia meningoseptica *[27]	0.063	7.5	119	73
*Spirosoma linguale*	1.1	173	163	100

*∗*Relative to *S. linguale*.

**Table 7:** *α*-NAGA conversion rate relating antigen number to turnover rate.

	Blood type	Antigen number	k_cat_ (s^−1^)	Conversion rate (Hr)
*E. meningosepticumα*-NAGA	A_1_	1.50E+06	10	42
	A_2_	250000	10	6.9
*S. lingualeα*-NAGA	A_1_	1.50E+06	173	2.4
	A_2_	250000	173	0.4

## Data Availability

The research data used to support the findings of this study are included within the article.
